# Primary Cardiac Angiosarcoma Diagnosed by Multimodality Imaging: A Case Report

**DOI:** 10.2174/0115734056352556250226080204

**Published:** 2025-03-17

**Authors:** Qin Zhang, Shuying Luo, Hua Ye, Tao Yang, Tijiang Zhang, Bangguo Li, Hong Yu

**Affiliations:** 1 Department of Radiology, Affiliated Hospital of Zunyi Medical University, Medical Imaging Center of Guizhou Province, Zunyi 563003, China; 2 Department of Pathology, Affiliated Hospital of Zunyi Medical University, Zunyi 563003, China; 3 Department of Ultrasound, Affiliated Hospital of Zunyi Medical University, Zunyi 563003, China

**Keywords:** Cardiac angiosarcoma, Right atrium, Multimodality imaging, Magnetic resonance imaging, Cardiac metastases, Computed Tomography

## Abstract

**Background:**

Primary cardiac tumors are rare. Most primary cardiac tumors are benign, with approximately 10.83% being malignant. We present a rare case of Primary Cardiac Angiosarcoma (PCA) with multiple metastases diagnosed using multimodality imaging, to enhance the understanding of PCA among clinicians and radiologists.

**Case Description:**

A 29-year-old woman presented to our hospital with a 2-day history of chest tightness, chest pain, palpitations, and dyspnea after physical activity. Ultrasonography and Computed Tomography (CT) of the heart revealed a mass in the right atrium. Cardiac magnetic resonance imaging suggested either a large cardiac lymphoma or angiosarcoma. The histopathological diagnosis confirmed a cardiac angiosarcoma. Positron Emission Tomography-Computed Tomography (PET/CT) revealed intense 18F-fluorodeoxyglucose (18F-FDG) uptake in the right side of the heart, with a maximum standardized uptake value of 10.9. Three months later, the patient was re-examined using abdominal CT, echocardiography, and PET/CT. PET/CT revealed increased 18F-FDG uptake which had become more extensive, with multifocal metastatic nodules in both the lungs and mediastinum. The patient was lost to follow-up after being discharged on May 1, 2022.

**Conclusion:**

The combined evaluation using multimodality imaging plays a vital role in determining the precise size and localization of the PCA, detecting distant metastases, and assessing patient prognosis.

## 1 INTRODUCTION

Primary Cardiac Angiosarcoma (PCA) is a highly aggressive malignant tumor that originates in the chamber or valve of the heart, most commonly in the right atrium and interatrial septum [1]. Its pathogenesis remains unclear. The clinical symptoms of PCA vary based on the tumor’s location, size, and degree of invasion and are often nonspecific, making early diagnosis challenging. The median overall survival of untreated patients is only 6 months [2]. Here, we present a case of PCA with multiple metastases to enhance the understanding of clinicians and radiologists and improve their ability to diagnose this rare condition.

## 2 CASE PRESENTATION

A 29-year-old woman was admitted to our hospital on December 31, 2021, because of symptoms of chest tightness, chest pain, palpitations, and dyspnea following activity for 2 days. Two days prior, the patient was diagnosed with pericarditis at another hospital; however, no significant improvement after symptomatic treatment was observed. She presented to our hospital for further diagnosis and treatment. Upon admission, her body temperature was 36.4°C, and her blood pressure was 140/99 mmHg. The patient was previously healthy and had no family history of any medical conditions.

Her laboratory test results revealed the following values: white blood cell count of 9.83×10^9^/L, neutrophils at 9.24×10^9^/L, lymphocytes at 0.49×10^9^/L, C-reactive protein level of 48.285 mg/L, N-terminal pro-B natriuretic peptide of 454 pg/mL, total protein determined in pericardial fluid samples at 63.9 g/L, lactate dehydrogenase, adenosine deaminase, and Nucleated Cell Count (NCC) was 957 U/L, 11.14 U/L, and 5696×10^6^/L, respectively. Additionally, the Rivalta test was positive, and the tumor marker CA-125 level was 55 U/mL.

**Fig. (1):**
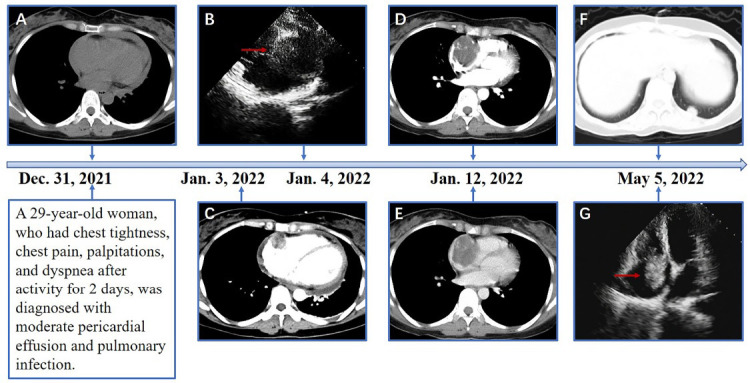
The timelines of this radiological examination. (**A**) The chest CT scan showed pericardial fluid and no obvious abnormalities concerning the heart. (**C**) Accidentally, an incidental abdominal CT with an enhancement scan suggested a filling defect in the right atrium. (**B**) The subsequent echocardiography revealed an isoechoic mass measuring 54mm × 42mm in the right atrium and right auricular region. (**D**, **E**) Arterial phase imaging and venous phase imaging revealed a marked inhomogeneous progressive enhancement of the right atrial mass with vascular shadows. (**F**) A lung window image showed a metastatic nodule in the lower left lung. (**G**) Echocardiography results of the reexamination revealed an isoechoic mass.

**Fig. (2):**
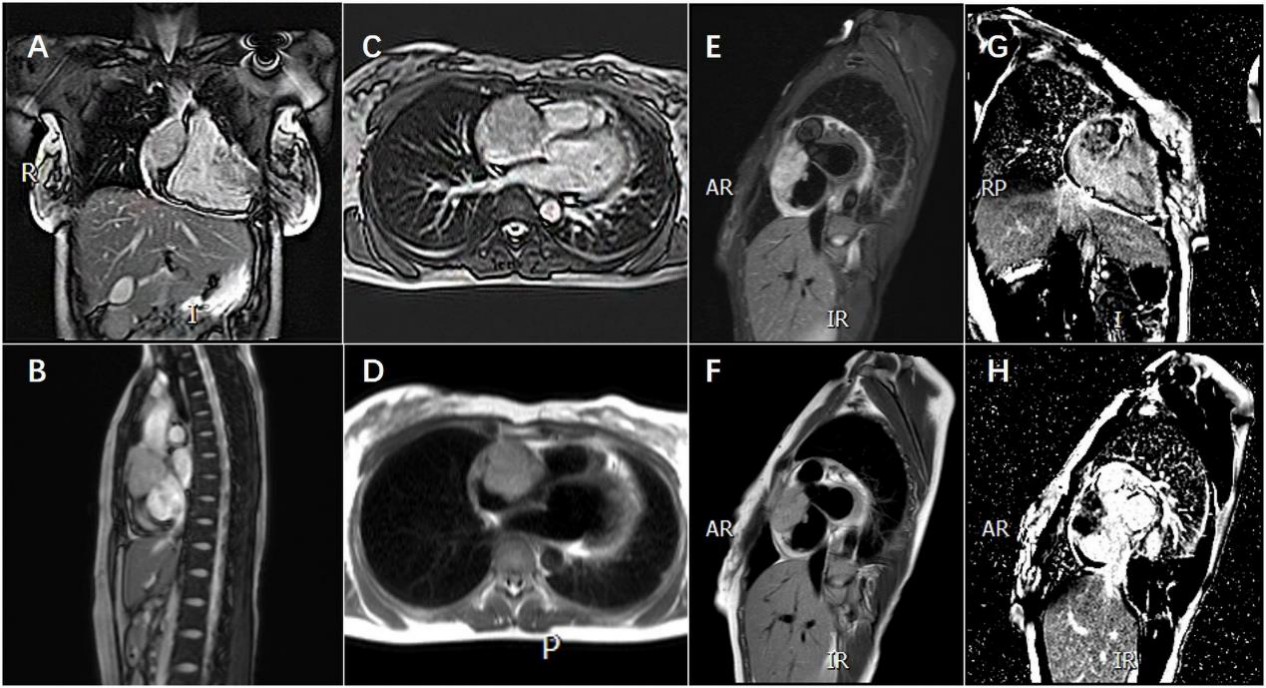
Different images of cardiac angiosarcoma during cardiac magnetic resonance imaging. (**A**-**C**) On bright-blood T2-weighted MRI, there was an iso intensity soft tissue mass. (**D**, **F**) On dark-blood T2-weighted MRI, four-chamber and short-axial view confirmed infiltration of the right atrium free wall and atrioventricular groove. (**E**) The mass showed hyperintense on the T2-weighted turbo inversion recovery magnitude (T2w-TIRM) sequence. (**G**, **H**) Short-axial late gadolinium enhancement (LGE) on CMR appeared to be a heterogeneous pattern of delayed enhancement.

**Fig. (3):**
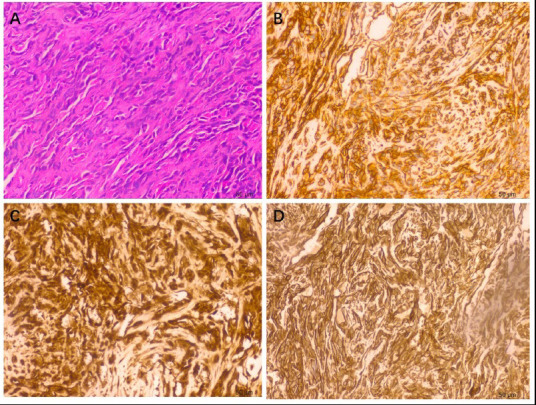
Histopathological image. (**A**) Hematoxylin and eosin staining revealed atypical spindle cells, original magnification ×20. Immunohistochemistry photomicrographs of CD34 (**B**), ERG (**C**), and Vimentin (**D**) stain, original magnification ×20; the tumor cells are positive for these markers.

**Fig. (4):**
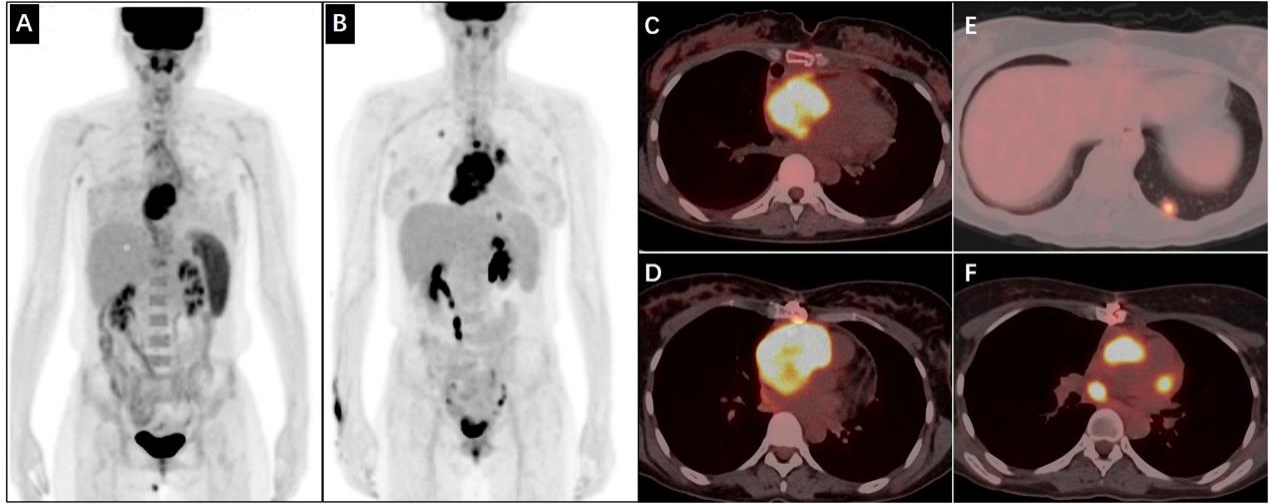
A ^18^F-FDG PET/CT scan. (**A**, **C**) Maximum intensity projection (MIP) image and fused PET/CT images revealed intense ^18^F-FDG uptake in the right side of the heart. The maximum standardized uptake value was 10.9. (**B**, **D**) MIP image and fused PET/CT images repeated after three months showed the increased ^18^F-FDG uptake had become more extensive, compared with A and C. In addition, multifocal ^18^F-FDG uptake was demonstrated in both lungs and mediastinum. (**E**, **F**) Multiple nodules with clearly increased ^18^F-FDG activity in the lungs and mediastinum were newly visualized.

Ultrasonography revealed moderate amounts of pericardial fluid. Chest Computed Tomography (CT) revealed mild pneumonia in the lower lobes of both lungs, moderate pericardial effusion, and no evident abnormalities in heart size, shape, or density (Fig. **1A**). Abdominal CT, extending to the lower chest level, suggested right atrial occupancy (Fig. **1C**). Cardiac Magnetic Resonance Imaging (MRI) revealed an irregular and heterogenous mass in the central and upper regions of the right atrium, exhibiting isointensity on T1-weighted images and hyperintensity on T2-weighted images. The mass invaded the right atrial wall and extended outward to the atrioventricular groove and pericardium, with a heterogeneous enhancement pattern on Late Gadolinium Enhancement (LGE) (Fig. **2**). Thus, it is reasonable to consider it a cardiac angiosarcoma or lymphoma. Subsequent echocardiography revealed an isoechoic mass spanning 54 × 42 mm in the right atrium and auricular region (Fig. **1B**). Chest contrast-enhanced CT revealed marked, inhomogeneous progressive enhancement of the right atrial mass with vascular shadows (Fig. **1D-E**). The patient then underwent an exploratory thoracotomy, where a fish-like tumor was observed in the right atrial appendage and ventricle. Complete resection was challenging due to the mass invading the superior and inferior vena cava and pericardium. A portion of the tumor was excised for pathology, and histopathological examination with hematoxylin-eosin staining revealed a proliferative spindle cell lesion within the right atrium with the following immunohistochemical results: Ki-67 (40%+), ERG (++++), CD34 (++++), CD31 (++++), SMA (+), Vimentin (++++), and Bcl-2 (weakly +). The pathological diagnosis was cardiac angiosarcoma (Fig. **3**). Four days after surgery, the patient underwent Positron Emission Tomography-Computed Tomography (PET/CT), which revealed an irregular soft tissue mass in the right atrium with 18F-fluorodeoxyglucose (18F-FDG) uptake and hypermetabolism (Fig. **4A, C**). The patient refused radiotherapy and chemotherapy but chose to be discharged and wait for a heart transplant. In May 2022, the patient was reexamined using echocardiography (Fig. **1G**), abdominal CT, and PET/CT. Echocardiography revealed a mass measuring approximately 48 × 34 mm within the right atrium. Furthermore, a hypoechoic lesion approximately 17 × 14 mm in size was observed in the right posterior-inferior region of the right atrium, adjacent to the entrance of the inferior vena cava. Abdominal CT revealed a high-density nodule measuring approximately 15 mm × 9 mm in the left lower lobe of the lung (Fig. **1F**). The PET/CT scan revealed that the right atrial mass exhibited an increase in size compared to that of the previous scan (Fig. **4B, D**) and also revealed multiple hypermetabolic nodules in both the lungs and mediastinum (Fig. **4E** - **F**). Regrettably, the patient was lost to follow-up, as we were unable to reach her after discharge on May 10, 2022, due to her phone number being disconnected.

## 3 DISCUSSION

A large-scale meta-analysis reported that primary malignant cardiac tumors account for approximately 10.83% of all primary cardiac tumors [3]. The most common types of primary malignant cardiac tumors are sarcomas, including angiosarcomas, rhabdomyosarcomas, fibrosarcomas, and Kaposi sarcomas. PCA is the most common type, accounting for approximately 30% of all malignant cardiac tumors [4]. It is characterized by the rapid formation of abnormal blood vessels that infiltrate the myocardium and cause damage to healthy heart tissue. The endothelial markers CD31 and CD34 are valuable for verifying whether a tumor originates from the endothelium. Low Ki-67 expression is associated with long-term survival [5]. Previous studies have shown that mutations in genes such as vascular endothelial growth factor receptor (VEGFR-2), protection of telomeres 1 (POT1), and other genes are present in patients with PCA [6]. The location of PCA is complex, clinical symptoms are nonspecific, and dyspnea is the most common symptom [7]. In this case, the PET/CT scan showed multiple hypermetabolic nodules in both the lungs and the mediastinum. These findings suggest that the tumor may already have metastasized to distant sites. This observation further supports the finding that the majority of PCA patients already present with distant metastases at the time of diagnosis [8].

PCA is most common in middle-aged adults aged 30–50, with a male-to-female ratio of approximately 3:1 [9]. Here, we report the case of a 29-year-old female with an angiosarcoma located in the right atrium. The patient presented with the typical symptoms of angiosarcoma, including chest tightness and dyspnea. Ultrasonography revealed a moderate amount of pericardial effusion. This finding is consistent with those of previous reports that identified pericardial effusion as the most common complication [10].

In this case, PCA and its associated complications were comprehensively evaluated using various imaging techniques, including echocardiography, CT, MRI, and PET/CT. This underscores the importance of increasing clinicians’ awareness of PCA. When young patients present with unexplained pericardial effusion, primary malignant cardiac tumors should be considered in the differential diagnosis, even if non-contrast chest CT scans reveal no significant abnormalities in the size, shape, or density of the heart. Multimodal imaging techniques are essential for comprehensive and accurate diagnostic evaluation.

The combined evaluation of multimodal imaging is crucial for the early and differential diagnosis of PCA. Echocardiography is non-invasive and convenient, enabling real-time assessment of cardiac structure, function, and blood flow dynamics. However, its clinical utility is limited by a narrow imaging window, leading to potential subjectivity and incomplete tumor evaluation. With its superior spatial resolution, CT offers precise visualization of tumor location, dimensions, density, and adjacent anatomical structure. Contrast-enhanced CT improves the differentiation of organs and tissues, enhancing the detection of abnormalities and lesions. Nevertheless, CT’s limited functional information and radiation exposure give CMR a distant advantage. CT often shows PCA as a homogeneous or inhomogeneous, low-attenuation, or isoattenuation mass on unenhanced scans, with rim enhancement or heterogeneous centripetal enhancement on enhanced scans [11]. For soft tissues, MRI provides remarkably superior resolution compared to other imaging modalities. MRI utilizes multiple sequences and metrics, offering exceptional resolution for soft tissue assessment. These advantages not only facilitate the diagnosis but also aid in the differential diagnosis of lesions. PCA often presents as heterogeneous or isointense on T1-weighted images and hyperintense on T2-weighted images. Owing to its propensity for hemorrhage, areas of PCA may exhibit increased signal intensity on T1-weighted images [12]. On MRI, PCA shows heterogeneous centripetal and rim enhancement, which are characteristic enhancement patterns. Fluorodeoxyglucose (FDG) uptake reflects the glycolytic activity of tumors, making 18F-FDG PET/CT a valuable tool for differentiating benign from malignant neoplasms. It also determines the extent of the primary tumor and detects the presence of distant metastases. However, this examination involves exposure to ionizing radiation and incurs substantial costs, which are barriers to its routine implementation and widespread adoption.

PCA should be distinguished from the following diseases: (1) Cardiac Lymphoma (CL): CL is typically located in the right heart, particularly the right atrium, and its principal clinical symptoms are dyspnea and pleural effusion. On MRI, CL appears as an isointense or hypointense mass on T1-weighted images. Conversely, T2-weighted images revealed marginally increased signal intensity due to diffuse edema. In contrast to CL, central necrosis is more frequently observed in cases of PCA, and the enhancement of PCA is more pronounced on CT or MRI scans [13]. (2) Cardiac Myxoma (CM): CM is the most common benign cardiac tumor in adults. It is often found in the left atrium and is attached to the fossa ovalis using a pedicle. Calcification, cystic degeneration, and necrosis may be observed. CT shows CM as a heterogeneous and hypodense mass. On MRI, CM is often depicted as a heterogeneous isointense mass on T1-weighted images and heterogeneous hyperintense mass on T2-weighted images. Echocardiography or CMR cine sequences vividly display the mobility of these lesions, which may even cause acute obstruction of the atrioventricular valve [14]. (3) Cardiac Hemangioma (CH): CH is an uncommon benign cardiac tumor composed of vascular engorgement channels lined by benign endothelial cells. Due to slow blood flow, CH often appears as isointense or hypointense on T1-weighted images and hyperintensity on T2-weighted images. With the prolongation of echo time, the contrast between the lesion and the surrounding normal tissues gradually increases (light bulb sign) [15].

One major limitation of this study was the lack of follow-up data. This limitation restricted our ability to thoroughly evaluate the long-term prognosis of patients. Future research should include comprehensive follow-up to better understand the outcomes of these patients.

## CONCLUSION

PAC represents an exceedingly aggressive malignancy characterized by atypical clinical manifestations, posing challenges for early detection and diagnosis. Distant metastases were found at the time of diagnosis in patients with PCA, leading to an unfavorable prognosis. Multimodality imaging is indispensable for precisely localizing the tumor, detecting distant metastases, and assessing the prognosis of patients with PCA. Despite advancements in imaging techniques, a biopsy remains the definitive method for establishing a diagnosis of PCA.

## Data Availability

Not applicable.

## LIST OF ABBREVIATIONS

CT: Computed Tomography
MRI: Magnetic Resonance Imaging
LGE: Late Gadolinium Enhancement
PET/CT: Positron Emission Tomography-Computed Tomo-graph
VEGFR-2: Vascular Endothelial Growth Factor Receptor 2
POT1: Protection of Telomeres 1
CL: Cardiac Lymphoma
CM: Cardiac Myxoma
CH: Cardiac Hemangioma
^18^F-FDG: ^18^F-fluorodeoxyglucose

## STANDARDS OF REPORTING

CARE guidelines were followed.
